# Burden of Latent Tuberculosis Infection (LTBI) Among Patients Attending a Tertiary Care Hospital in Eastern India: A Descriptive Study

**DOI:** 10.7759/cureus.104511

**Published:** 2026-03-01

**Authors:** Aditya Kundu, Diptanu Majumder, Debjani Das, Niladri Das

**Affiliations:** 1 Microbiology, Manipal Tata Medical College (MTMC), Manipal Academy of Higher Education, Jamshedpur, IND; 2 Microbiology, All India Institute of Medical Sciences, Kalyani, Kalyani, IND

**Keywords:** autoimmune diseases, igra, ltbi, tb, tb elimination

## Abstract

Introduction: Latent tuberculosis infection (LTBI) is still neglected and acts as a barrier to the tuberculosis (TB) elimination goal of the National Tuberculosis Elimination Programme (NTEP) by 2025 sustaining TB transmission, particularly in high-burden countries like India. Unlike active TB, which is symptomatic and contagious, LTBI is non-infectious and asymptomatic. However, in LTBI, *Mycobacterium tuberculosis* (*M. tuberculosis*) bacilli can reactivate and cause disease under favorable conditions, thus forming a reservoir for future TB cases.

Materials and methods: This hospital-based observational descriptive retrospective study was conducted at a tertiary care center in Eastern India from November 2024 to April 2025. The study included adult patients (>18 years) who were referred for LTBI screening using interferon-gamma release assay (IGRA) during the study period. A total of 236 patients were analyzed. Demographic and clinical details, including age, sex, residence (urban/rural), comorbidities, history of immunosuppressive therapy, documented TB exposure, and other risk factors, were extracted from patient case files and electronic hospital records using a structured data collection format.

Results: LTBI was detected in 56 patients, yielding a prevalence of 23.7% (56/236). Most LTBI-positive individuals were male and aged 30-45 years. A high proportion had underlying autoimmune diseases, while 19.6% (11/56) had no identifiable risk factors which refers to the absence of documented traditional exposures (e.g., household contact), comorbid condition, or clinical indication for screening.

Discussion: The substantial LTBI burden, particularly among immunocompromised individuals, highlights gaps in targeted screening strategies. Our study identified the proportion of LTBI among patients attending a tertiary care hospital in Eastern India to be 23.7% which is lower than the global average of about 33%. The relatively younger and healthy population participating in the study can also contribute to the low LTBI frequency, as older individuals usually have a high cumulative risk of *M. tuberculosis*. Although very specific, the use of a single clinical method like IGRA might have led to the sub-detection of LTBI or false-negative results, especially in immunosuppressed individuals. One of the most striking conclusions was the high ratio (71.4%) of LTBI-positive individuals with underlying autoimmune disease. It corresponds to the existing literature, which exposes the increasing sensitivity of this population to TB, especially when one goes through immune stress therapy such as corticosteroids, tumor necrosis factor-alpha (TNF-α) inhibitors, etc.

Conclusion: LTBI represents a silent threat to TB control. Patients with autoimmune disease represent a group at high risk, especially on immunosuppressive medications. A substantial proportion of LTBI-positive individuals had no identifiable risk factors, suggesting that relying solely on known exposure history or symptom-based screening may miss a significant number of infections. Expanded screening using advanced monitoring, modern high-risk diagnosis, and preventive therapy should be an integral part of the national reaction to TB.

## Introduction

Despite the ongoing efforts of the National Tuberculosis Elimination Program (NTEP), the goal to eliminate tuberculosis (TB) as a public health problem by 2025 in India is not achievable due to various factors, and globally, India contributes 26% of TB cases. Approximately 25-30% of the world's population is estimated to be infected with *Mycobacterium tuberculosis* (*M. tuberculosis*) in latent form. This corresponds to about 2-2.3 billion people globally [1]. Latent tuberculosis infection (LTBI) diagnosis and management is an important and neglected factor which is causing a barrier in TB elimination. Unlike active TB, which is symptomatic and contagious, LTBI is non-infectious and asymptomatic. However, in LTBI, *M. tuberculosis* bacilli can reactivate and cause disease under favorable conditions, thus forming a reservoir for future TB cases.

LTBI is defined as a persistent immune response to TB antigen in the absence of clinical symptoms or radiographic evidence of active TB [2]. It is estimated that about a third of the world's population, 2.3 billion people, are infected with TB [2,3]. Although only 5-10% of people with LTBI develop active TB during life, this risk increases significantly in immunocompromised individuals, such as those with HIV, diabetes, or autoimmune conditions, as well as older and malnourished populations [4,5].

Clinical progress has improved the ability to detect LTBI more accurately. Traditional tuberculin skin tests (TST), although still in use, have several limits due to cross-reactivity with the Bacillus Calmette-Guérin (BCG) vaccine and non-tubercular *Mycobacterium* like *Mycobacterium avium* and *Mycobacterium intracellulare* [6]. Contrary to this, interferon-gamma release assay (IGRA) uses *M. tuberculosis*-specific antigens such as early secreted antigenic target of 6 kDa (ESAT-6), culture filtrate protein-10 (CFP-10), and TB 7.7 which has high specificity and less chance of cross-reactivity [7,8].

Despite these, LTBI is a low-priority subject in India's agenda for public health [9]. Work on monitoring and treatment has mainly focused on active TB cases. However, ignoring LTBI will prolong elimination goals. In addition, India has limited data on LTBIs' prevalence, especially among people with weak immunity such as those with autoimmune diseases, cancer patients, or individuals with long-term immunosuppressive therapy. Hence, this study was done to estimate the prevalence of LTBI among patients attending the hospital and to identify the demographic and clinical risk factors of LTBI. 

## Materials and methods

Study design and setting

This was a retrospective study conducted at the Department of Microbiology, All India Institute of Medical Sciences (AIIMS), Kalyani, a tertiary care institution located in Eastern India. The study duration spanned from November 2024 to April 2025.

The study population included individuals attending AIIMS, Kalyani, suspected of LTBI. Inclusion criteria involved adult patients (>18 years) with suspected LTBI referred for screening. Considering a pilot study, we have included all the patients who came to us for LTBI screening; a total of 236 patients were enrolled. Sociodemographic and clinical data, including age, sex, residence (urban/rural), comorbidities (particularly autoimmune diseases), and known TB exposure, were recorded using a structured data extraction proforma. Exclusion criteria involved patients with evidence of active TB, individuals with a previous history of treated or untreated active TB, patients currently receiving anti-tubercular therapy (ATT), and patients with incomplete clinical or laboratory records.

The IGRA test was performed using the STANDARD E TB-Feron enzyme-linked immunosorbent assay (ELISA) kit manufactured by SD Biosensor, Suwon-si, Republic of Korea, to detect LTBI. The cutoff, intermediate result, and quality control were validated as per the kit instructions. The method involves the collection of whole blood into three types of tubes: Nil control which measures baseline interferon-gamma (IFN-γ) levels, TB antigen tube which contains *M. tuberculosis*-specific antigens, and mitogen control which serves as a positive control to confirm immune response capability. Tubes were incubated at 37°C for 16-24 hours. ELISA analysis was done by the following procedure: plasma was harvested and added to a 96-well plate pre-coated with anti-IFN-γ antibodies. A biotinylated detection antibody and enzyme conjugate were sequentially added. A substrate was introduced to initiate a colorimetric reaction, proportional to IFN-γ concentration. Quantification was performed using SD Biosensor ELISA report software. A Microsoft Excel sheet (Microsoft, Redmond, WA, USA) was used to analyze the data. Records with incomplete key variables were excluded from analysis. Complete case analysis was performed, and no imputation of missing data was undertaken due to the retrospective nature of the study. Accumulated data were analyzed using descriptive statistics as frequencies and percentages, and results were described using tables and figures.

## Results

**Figure 1:**
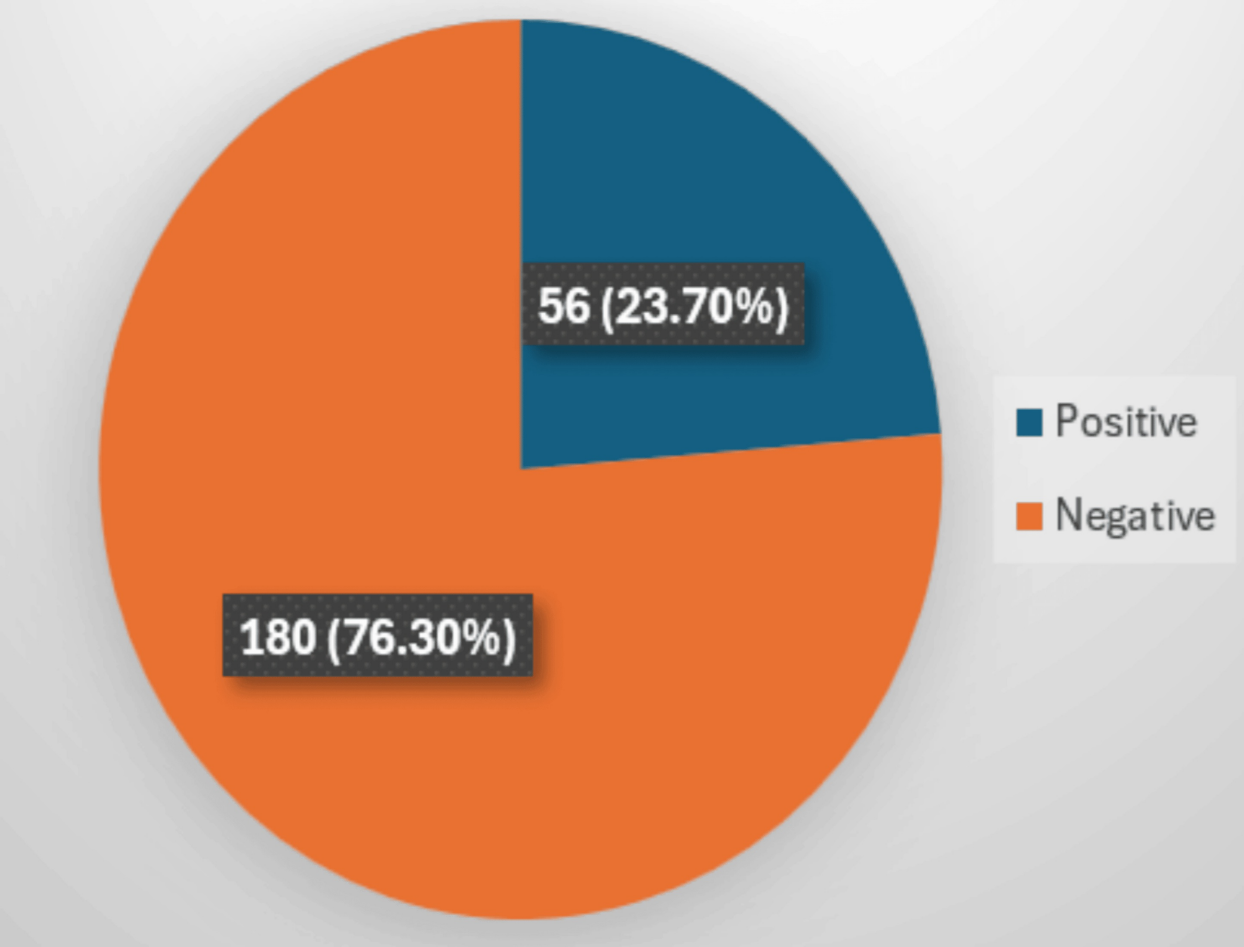
Distribution of latent TB TB: tuberculosis

Out of 236 patients tested, 23.7% (56/236) tested positive for LTBI using IGRA which is shown in Figure 1. About 80% (45/56) of the IGRA-positive patients were male. The 30-45-year age group constituted the most LTBI-positive cases, 33.92% (19/56), shown in Table 1. A slightly higher prevalence was observed among urban residents compared to rural counterparts (57.16% (32/56)).

**Table 1:** Age-wise distribution of latent TB TB: tuberculosis

Age groups	Total patient samples
1-15 years	5
16-30 years	16
31-45 years	19
46-60 years	9
61-75 years	7

**Figure 2:**
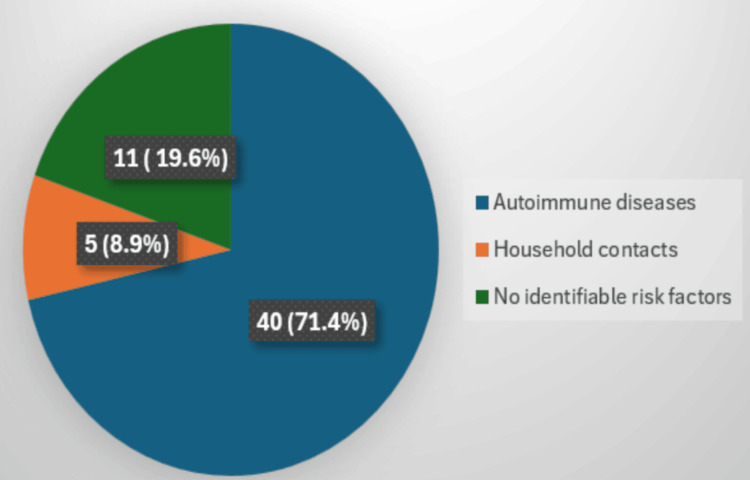
Risk factors associated with latent TB TB: tuberculosis

Among the 56 IGRA-positive individuals, 71.4% (40/56) had underlying autoimmune diseases, including rheumatoid arthritis (RA), systemic lupus erythematosus (SLE), and inflammatory bowel disease (IBD). Around 8.9% (5/56) reported close household contact with confirmed TB cases. About 19.6% (11/56) had no identifiable risk factors, highlighting the role of subclinical or unknown exposures shown in Figure 2.

## Discussion

Our study identified the proportion of LTBI among patients attending a tertiary care hospital in Eastern India to be 23.7% which is lower than the global average of about 33% [2,3]. However, it is important to explain this in light of the study population characteristics. Unlike global figures, often derived from large-scale community-based studies, our study was based on a targeted clinical population, which was composed of symptomatic or high-risk individuals referred to for screening. Therefore, although the circulation may appear less, it still indicates a significant load of latent infection in specific groups which is unsafe for the TB progress.

Many factors can explain this lower-to-global circulation. Regional variations are expected in TB transmission speeds, as TB phenomena are different in different Indian states and communities [10,11]. The relatively younger and healthy population participating in the study can also contribute to the low LTBI frequency, as older individuals usually have a high cumulative risk of *M. tuberculosis*. Although very specific, the use of a single clinical method like IGRA might have led to the sub-detection of LTBI or false-negative results, especially in immunosuppressed individuals [12]. The findings from our study on LTBI provide many important insights, especially in the context of India's ongoing struggle to eliminate TB.

One of the most striking conclusions was the high ratio (71.4%) of LTBI-positive individuals with underlying autoimmune disease [4,5]. It corresponds to the existing literature, which exposes the increasing sensitivity of this population to TB, especially when one goes through immune stress therapy such as corticosteroids, tumor necrosis factor-alpha (TNF-α) inhibitors, etc. This strong association emphasizes the requirement of regular LTBI screening in autoimmune patients, especially before starting immunosuppressive treatment. It is consistent with the findings by Feuchtenberger et al., which reported the importance of screening practice with TST and IGRA in RA patients [13,14]. Integration of LTBI diagnostics into the clinical workflow for autoimmune patients can significantly reduce the chance of latent TB in this subgroup.

About 8.9% reported prior household contact with a confirmed TB case. As this study did not include inferential statistical analysis to examine associations between exposure variables and LTBI positivity, no conclusions can be drawn regarding the strength or significance of household contact as a risk factor in our study population, while domestic risk is a well-established risk factor due to long-lasting close contact. Further analytical studies with appropriate statistical testing are required to evaluate the relationship between domestic TB exposure and LTBI. It is also possible that community-based transfer, rather than domestic risk, plays a more important role in LTBI transmission [15].

However, the association confirms the importance of contact tracing and education on TB transmission in family settings. It emphasizes the LTBI detection and supports preventive strategies for domestic contacts in active TB cases.

Approximately one-fifth of IGRA-positive individuals had no documented traditional risk factors such as known household TB contact or identifiable comorbidities. This emphasizes the frequent cryptic nature of TB transmission, which can occur through a random social interaction or the result of the transfer of old infections acquired in childhood or adolescence [11]. While this finding does not establish the absence of association, it may reflect unrecognized exposures, incomplete documentation, or community-level transmission in high-burden settings. Further analytical studies with multivariable assessment are needed to clarify the independent risk determinants of LTBI in this population.

In high-bound countries such as India, this discovery supports logic for broad, population-based, or risk-independent screening strategies. Such perspectives will help to identify latent infections, especially in people without classical risk factors requiring preventive therapy.

Important demographic trends were also detected in our data. Most LTBI-positive individuals were in the 30-45-year age group. This may be caused by an increase in commercial risk, especially in workplaces in the overload or under densely populated urban areas [10].

The difference between the urban and countryside can be attributed to several factors, including higher population density, air pollution, and overcrowded living or working conditions, all compatible with the air transfer of TB. The urban environment often creates micro-epidemics where the transfer continues quietly, especially with limited access to health services in the slum and informal residential areas [10,11].

These findings require more long-awaited public health interventions that consider unique challenges with urban environments, including targeted screening and community-based seeking in high-risk areas.

Limitation of the study

This study is retrospective in nature, and a single tertiary care center was involved which may limit the generalizability of the results to the wider community. Particularly in immunocompromised patients, the use of IGRA alone without TST or radiological follow-up may underestimate LTBI. Moreover, the absence of longitudinal follow-up barred the assessment of progression from LTBI to active TB.

## Conclusions

LTBI represents a hidden epidemic in a wide TB landscape. In this hospital-based study, 23.7% of individuals screened were IGRA positive. A substantial proportion of LTBI-positive cases had underlying autoimmune disease; however, given the descriptive design of this study, independent risk associations cannot be established. LTBI is an important quiet threat to TB control, able to detect the active TB case and reverse the benefits received in the treatment. Patients with autoimmune disease represent a group at high risk, especially on immunosuppressive medications. A substantial proportion of LTBI-positive individuals had no identifiable risk factors, suggesting that relying solely on known exposure history or symptom-based screening may miss a significant number of infections. These findings highlight the importance of structured LTBI screening among high-risk clinical groups in tertiary care settings. However, larger multicentric and population-based studies are required before broader policy-level recommendations can be made. Only then can India expect to break the transmission cycle and get closer to the TB-free future goal.
